# Investigating the Norwegian eHealth Governance Model: Document Study

**DOI:** 10.2196/59717

**Published:** 2024-12-04

**Authors:** Line Helen Linstad, Hilde Bjørnå, Anne Moen, Truls Tunby Kristiansen, Anne Helen Hansen

**Affiliations:** 1 Department of Community Medicine Faculty of Health Sciences UiT The Arctic University of Norway Tromsø Norway; 2 Norwegian Centre for E-health Research Tromso Norway; 3 Department of Social Sciences Faculty of Humanities, Social Sciences and Education UiT the Arctic University of Norway Tromsø Norway; 4 Department of Public Health Sciences Institute of Health and Society University of Oslo Oslo Norway; 5 University Hospital of North Norway Tromsø Norway

**Keywords:** eHealth policy, fragmented decision authority, top-down governance, bottom-up network, participation, electronic health record

## Abstract

**Background:**

Governments and policy makers struggle to achieve a balance between hierarchical steering and horizontal governance in systems characterized by fragmented decision authority and multiple interests. To realize its One Citizen–One Journal eHealth policy vision, the Norwegian government established a special eHealth board of stakeholders to create an inclusive governance model that aligned stakeholders’ interests with the government’s ambitions through coordination and consensus. Little empirical knowledge exists on how countries realize inclusive governance models.

**Objective:**

This study aims to investigate how the Norwegian inclusive eHealth governance model was developed as a tool to align the government’s policy ambitions with stakeholders’ concerns from January 2012 to December 2022.

**Methods:**

This document study used a thematic analysis based on a constructivist research approach. We included 16 policy documents and 175 consultation response documents issued between January 2012 and December 2022 related to the Norwegian One Citizen–One Journal policy implementation process. The themes were constructed deductively from a review of governance models and public governance theory and were applied as our analytical lens to each document. The findings were interpreted, analyzed, and synthesized.

**Results:**

The national policy implementation process progressed through 3 phases, with changes in stakeholder inclusion and perceived influence on the decision-making process characterizing transitions from phase to phase. Tension developed between 2 contrasting views regarding top-down government authority and stakeholders’ autonomy. The view of the regional health trusts, municipalities, health care professional organizations, and industry actors contrasted with that of the patient organizations. Governmental insensitivity to participation, lack of transparency, and decreasing trust by stakeholder groups challenged the legitimacy of the inclusive governance model.

**Conclusions:**

We illustrated that Norway’s One Citizen–One Journal policy trajectory was characterized by a process that unfolded across 3 distinct phases. The process was characterized by 2 contrasting stakeholder perspectives. Finally, it was shaped by diminishing trust in the inclusive governance model. The National eHealth Governance Board faced challenges in establishing legitimacy as a top-down inclusive governance model, primarily attributed to its addressing of participation, transparency, and trust dilemmas. Such dilemmas represent significant obstacles to inclusive governance models and require ongoing governmental vigilance and responsiveness from governmental entities.

## Introduction

### Governance Models in a Multistakeholder Field

The successful implementation of national eHealth programs requires countries to be sensitive to the dynamics of governance and participation [1]. Countries struggle to achieve a balance between hierarchical steering and horizontal governance to create inclusive governance models that nurture synergy among all stakeholders involved in the adoption of eHealth in national health care systems [1,2]. The inclusiveness of the model relates to how it enables stakeholder participation in the policy implementation process [3].

eHealth is a multistakeholder field in which numerous self-regulated actors participate, representing government bodies, health care services, health care professionals’ labor unions, industry vendors, and citizens (patients, next of kin, and their interest organizations) [4,5]. These stakeholders have diverse interests, fragmented decision-making authority, and varying operating logics, but they all endorse the same health policy goal, that is, to realize patient-oriented, digitally supported health services across organizational borders [6,7].

Few empirical studies have considered how countries realize inclusive governance models to align stakeholders’ interests [2]. Through a recent literature review of eHealth governance models, we found that policy processes and goal attainment often result from negotiations between governments and self-regulated actors [2,8]. The review also revealed that such negotiations generate governance dynamics among hierarchical steering, medical bottom-up governance, market governance, and patients’ concerns about their health data and participation in health-related procurement decisions [9].

Insights from Denmark and New Zealand have indicated that top-down policies are often met with bottom-up reactions, prompting negotiation processes [1,2,10,11]. The United Kingdom’s and Denmark’s experiences have demonstrated that eHealth processes are complex and unpredictable, involving various stakeholders [1,2,11,12]. To maintain progress, researchers have proposed stronger hierarchical steering of the sector to ensure increasing digitalization and innovation in health care [13]. In Scandinavia, eHealth policies are characterized by unstable governance structures, as illustrated by the Danish government’s back-and-forth changes in policy, from top-down governance to horizontal governance [1,2,11].

### Norwegian Context

The challenges of governing the fragmented eHealth sector, comprising self-regulated actors’ diverse interests, are evident in Norway [4,14]. In 2012, the government introduced its eHealth vision, encapsulated in the concept of One Citizen–One Journal (OCOJ) as the overarching objective. The OCOJ initiative aims to address three distinct sets of stakeholder needs: (1) ensuring that health care personnel have easy and secure access to patient data, (2) providing citizens with easy and secure access to digital health services, and (3) making data accessible for quality improvement, health monitoring, governance, and research purposes [4].

The government described eHealth as a domain with a plethora of stakeholders exhibiting different decision authorities and interests. From the government’s point of view, the OCOJ was seen as a response to the stakeholders’ articulated need for a stronger top-down approach in eHealth [4]. The white paper mandated the Ministry of Health and Social Care to outline the implementation of a national electronic health record (EHR) in a report [4,15].

**Figure 1:**
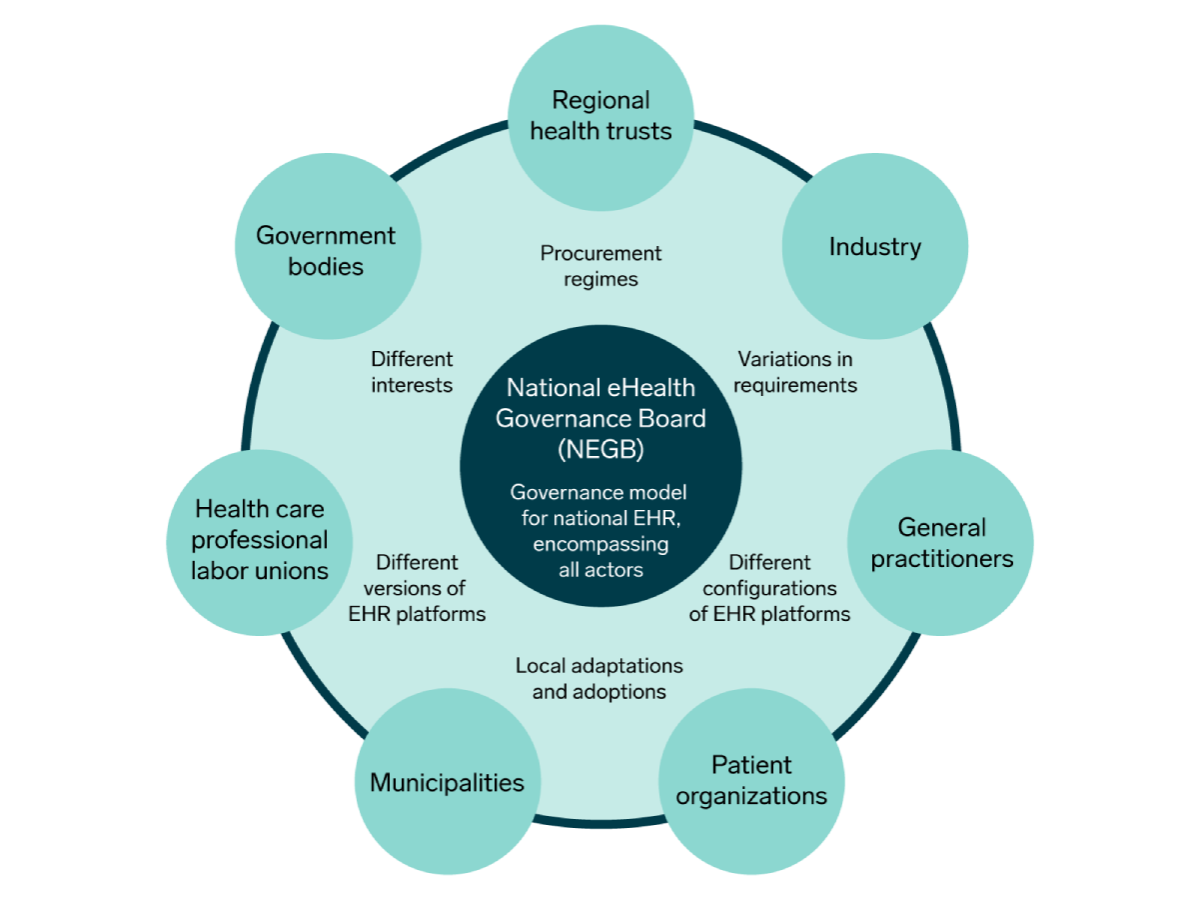
The Norwegian eHealth Governance Board and the stakeholder groups wanting to be board members and participate in the policy implementation process. EHR: electronic health record.

Norway’s strategy was to create the National eHealth Governance Board (NEGB) to implement an inclusive national governance model. It comprised regional health trusts, municipalities, general practitioners (GPs), patient organizations, labor unions for health care professionals, and governmental bodies [15] (Figure 1). The board aimed to ensure that EHR policy implementation was realized via “a strong national governance model” grounded in consensus and coordination that could solve problems involving different interests; procurement regimes; variations in requirements; local adaptations and adoptions; and different versions, configurations, and EHR technological platforms [4,14]. Industry vendors were not included in the NEGB [4].

In 2016, the government established the Directorate of eHealth, the main purpose of which was to focus efforts on realizing and strengthening the OCOJ vision. The Directorate of eHealth acted as a secretariat for the NEGB [15]. Alongside the NEGB, the government proposed new eHealth regulations (eHealth Act 2019-2020) and a national funding scheme [14,16]. The proposed eHealth Act was retracted from the Norwegian Parliament by the end of 2020 following significant discussion of its implications [16].

The NEGB members were self-regulated actors bringing multiple voices and interests into the process that included the voices of the regional health trusts, the municipalities, the health care professionals, the patient organizations, and governmental bodies. In addition, the Norwegian Association of Local and Regional Authorities (KS) represented Norwegian municipalities in the NEGB (Figure 1).

Under the Norwegian constitution, municipalities are self-governed entities that align with the European Charter of Local Self-Government [17,18]. The municipalities are responsible for primary health care services, including managing agreements with self-governed GPs, while the state owns and operates specialist health care services in regional health care trusts [19-21]. Municipalities, GPs, and regional health trusts are responsible for investing in and implementing their EHR systems. The government and the municipalities meet twice a year under a consultation scheme to discuss policy implications for municipalities regarding the national budget and other policy issues. They have a meeting specifically related to digitalization policy once a year [22].

### Theoretical Perspectives: From Government to Governance

The term government refers to the formal institutions of the state that have a monopoly on legitimate hierarchical steering power [23,24]. In the 1970s and 1980s public governance literature, the perspective changed from a “hierarchical government paradigm” to a “horizontal governance paradigm” [23]. In the horizontal governance paradigm, the government “is not the only actor that attempts to inﬂuence societal developments, and government interventions are interventions in policy networks, in which power, resource dependency, and strategic behavior are vital elements” [23]. This implies that government interventions must negotiate multiple interests and adjust to stakeholders’ actions and reactions [24,25]. Successful government interventions based on inclusive national governance models depend on how the models facilitate alignment and foster inclusion of stakeholders’ concerns and top-down government ambitions in a multistakeholder environment [26,27].

These processes, in which the government and stakeholders collaborate to achieve a common goal within an inclusive governance model, may progress through distinct phases [28]. Governance models are constructions characterized by a mix of top-down governance, bottom-up network, and market governance [29]. In one phase, the model is predominantly characterized by an emphasis on the network approach. Conversely, in another phase, both the government and stakeholders may recognize the need for models that incorporate hierarchical structures or market-oriented strategies [29]. Recently, researchers in governance theory have questioned how the governance models must adapt to a global context of increased turbulence through a combination of network, market, and top-down governance [29-31].

We aligned our perspective on governance with “the nonhierarchical process whereby public and private actors and resources are coordinated and given common sense and meaning” [26]*.* Political goals result from negotiated compromises between the government and stakeholders [23,32,33]. Such negotiations are consequences of the inclusive governance model’s ability to address governance dilemmas and balance stakeholder concerns and top-down government ambitions [23,28]. These dilemmas may relate to issues such as participation, transparency, trust, mutual rules, governance processes, and stakeholders’ autonomy versus top-down government steering [2,23,27,34]. Governments may create inclusive top-down horizontal governance models to ensure that stakeholders’ interests and government policy goals are aligned [3,23]. However, inclusive horizontal governance may also emerge from bottom-up networks based on user needs [2,35].

To establish a robust model for trust management, stakeholders must perceive that their involvement in policy-making processes is meaningful, their concerns are acknowledged and addressed effectively, and they can influence decision-making outcomes [26,27,31]. Participation and transparency are vital for building and maintaining trust in governance models, and stakeholders’ participation in public governance models should be voluntary [23,27,36,37]. When stakeholders perceive that the government is using a hierarchical approach characterized by stringent, directive policies, trust levels are likely to diminish. Consequently, concerns may arise about inclusive governance models’ legitimacy, prompting questions about their efficacy and ability to accommodate diverse stakeholder perspectives [3,23,24]. This may lead to stakeholders withdrawing from the model or reducing their participation. However, if the government adopts a hands-off strategy, the model may be viewed as a loose network that allows members to cherry-pick policy themes and avoid difficult negotiations [23]. An inclusive horizontal governance model must address this dilemma to ensure stakeholder alignment and policy goal attainment [3,23]. To balance an inclusive horizontal governance model, the government may also increase its trustworthiness through mutually agreed-upon conflict-resolution procedures [3,23].

Through this study on the Norwegian eHealth governance model, we develop knowledge that is valuable for eHealth policy makers and stakeholders in practice. Furthermore, we aspire to stimulate increased multistakeholder debates on governance models in eHealth policy. We also aspire to contribute knowledge to eHealth policy research.

### Aims

This study aimed to investigate how the inclusive Norwegian eHealth governance model served as a policy tool to realize the Norwegian EHR policy, as outlined in the vision for OCOJ. Specifically, we analyzed how the governance model addressed public governance dilemmas by asking the following research questions: (1) What characterized national governance in the process of implementing the OCOJ policy in Norway from January 2012 to December 2022? and (2) How did the national governance model address public governance dilemmas by aligning multiple interests during different phases of the policy process in a fragmented field?

## Methods

### A Case Study With a Longitudinal Approach

This paper is based on a descriptive and interpretative case study conducted to analyze a major national eHealth policy implementation process [38,39]. We decided to perform a document study with a thematic constructivist approach [40]. This technique is commonly used in political science when analyzing policy processes and how they evolve [41]. By including policy documents from the government and consultation response documents we were able to uncover the most prominent policy themes. This approach facilitated a comparison of the government’s policy ambitions and the stakeholders’ perspectives on the different themes. Each document was treated as an “informant,” the same way as documents derived from transcribed qualitative interviews [42].

Analysis was performed between May 1, 2022, and June 1, 2023. Through a thick description of what the government and the stakeholders presented as their ambitions and perspectives, we established a detailed description of their social reality. The aim of applying a thick description may simplify other researchers’ assessment of our study’s compatibility with their context [40].

This paper provides an in-depth analysis; discusses its empirical findings and concepts, such as horizontal governance, hierarchical steering, and eHealth governance models; and presents analytical and conceptual generalizations [43].

### Search Strategy to Identify Empirical Material

To identify relevant documents from the OCOJ process, such as government policy documents and different stakeholders’ consultation response documents, we searched various websites to locate publicly available information issued by the Norwegian Parliament, the Ministry of Health and Social Care, and the Directorate of eHealth. We conducted our document search from May 1 to June 30, 2022. The search started on the Directorate of eHealth’s website, where we found a chronological list of all documents related to the OCOJ policy process. To identify relevant consultation response documents, we then searched the websites of the NEGB members and 2 non-NEGB umbrella organizations for industry vendors. We extended our search to the websites of the Ministry of Health and Social Care and the Norwegian Parliament, through which we accessed all consultation response documents from official hearings involving stakeholders on our topic of interest. We confirmed our search findings and inclusions by email correspondence with the Ministry of Health and Social Care, the Norwegian Parliament, the Directorate of eHealth, and KS. All documents are included in Multimedia Appendix 1.

### Inclusion and Exclusion Criteria

We resolved any disagreements through dialogue and consideration of the research questions. All documents directly related to OCOJ were included, along with those addressing policy issues related to national eHealth governance and consultancy reports commissioned by the government.

We excluded documents not written in Norwegian, policy documents on national health care reforms and general health policy, and documents on policy topics not concerned with horizontal eHealth governance and network governance. Interviews in newspapers, position documents, and policy notes on different stakeholders’ websites were not included. Furthermore, such documents were often comments on already-included documents. All included documents are listed in Table S1 in Multimedia Appendix 1.

Policy documents represent the government’s future visions. They are political tools to enable policy implementation processes that entail policy discussions, negotiations, and compromises between the government and the stakeholders. The consultation response documents reflect the policy perspectives of various stakeholders, including patients, medical professionals, industry representatives, and municipal authorities.

We have listed the report from the Office of the Auditor General of Norway under policy documents. This document may differ slightly from other policy documents as it aims to present an audit of the OCOJ policy implementation. We have chosen to list it under policy documents because we define the general auditor of Norway as a governmental actor and not as a stakeholder. Table 1 lists all policy documents included in the study, and Table 2 lists all included consultation documents.

Prop 3 L: Proposition to the Parliament for a decision on a legal act.

OCOJ: One Citizen–One Journal.

**Table 1:** Policy documents included in the study.

Period	Type of document	Author	Policy documents (N=16), n (%)
2012-2013	White Paper Number 9 (2012-2013): “One Citizen–One Journal” (OCOJ) [4]	Ministry of Health and Social Services	1 (6)
2013-2019	Reports on OCOJ [15,44-51]	Directorate of eHealth	9 (56)
2018-2020	Consultancy reports on OCOJ [52,53]	Holte Consulting	2 (12)
2019	eHealth Act, Prop.^a^ 65 L (2019-2020) A proposal to Parliament [16]	Ministry of Health and Social Services	1 (6)
2021	Office of the Auditor General of Norway [14]	An investigation conducted by the Office of the Auditor General of Norway on the Ministry of Health and Care Services’ governance of the work on OCOJ	1 (6)
2021	Changes in the Patient Journal Act and access to and payment for eHealth solutions and more: Prop 3 L (2021-2022), Innst^b^ 47 L (2021-2022), and Lovvedtak 26 (2021-2022) [54].	Ministry of Health and Social Services	1 (6)
2022	KS^c^ policy report [55]	The municipal sector’s eHealth ambitions	1 (6)

^a^Prop.: Proposition to the Norwegian Parliament for a decision on a legal act.

^b^Innst: A legal act proposal from one committee in the Norwegian Parliament to a plenary decision in the Parliament.

^c^KS: Norwegian Association of Local and Regional Authorities.

**Table 2:** Consultation response documents included in the study.

Date	Consultation response documents linked to [4,56]	Consultation response documents (N=175), n (%)
November 2012	White Paper Number 9 (2012-2013): OCOJ^a^	5 (3)
April 2019	eHealth Act, Proposal 65 law (2019-2020) Proposals to Parliament	91 (52)
October 2020	eHealth Act hearings in Norwegian Parliament	20 (11)
October 2021	Changes in the Patient Journal Act and access to and payment for eHealth solutions and more: Proposal 3 L (2021-2022), Innst^b^ 47 L (2021-2022), and Lovvedtak 26 (2021-2022)	59 (34)

^a^OCOJ: One Citizen–One Journal.

^b^Innst: a legal act proposal from one committee in the Parliament to a plenary decision in the Parliament.

### Search Terms

We constructed our search terms from public governance theory and a review of eHealth governance models. Textbox 1 presents an overview of search terms and their source (theory or review).

**Search terms and their source.**

**Ekeland and Linstad [2]**

- Top-down governance model
- Hierarchical governance model
- Bottom-up network governance model
- Bottom-up medical network governance model

**Ansell and Torfing [23]**

- Inclusive governance model
- Participation

**Klijn [25]**

- Network governance or horizontal governance

**Christensen [34]**

- Transparency

**Lane and Bachmann [37]**

- Trust

**Ansell and Torfing [23]**

- Governance dilemma

### Analysis

We analyzed the collected documents using a thematic document analysis from an analytical perspective based on governance perspectives from political science and a review of eHealth governance models (Textbox 1). The thematic analysis seeks to identify, analyze, organize, describe, and report themes found within a dataset [57]. This explorative thematic study entailed an iterative process of moving back and forth between the data and the theoretical perspective.

The review process was conducted manually by LHL, TTK, and AM. AHH and HB reviewed the final table and provided their input. LHL individually reviewed each included document (PDF files) and applied all search terms from Textbox 1 to each document. Relevant text matching the search terms was extracted and copied into a Microsoft Excel file alongside the corresponding document. Upon completion of this initial data extraction, TTK reviewed the data and engaged in discussions with LHL. Subsequently, LHL synthesized the findings from each document and compiled them into a table (Microsoft Word file). TTK reviewed the table and provided feedback. AM then reviewed both the data and the synthesized findings from each document. LHL and AM discussed and reached a consensus on the text and the level of synthesis. The table of documents is included in Table S1 in Multimedia Appendix 1.

Through this data extraction and subsequent synthesis of data, we were able to elicit the thematic OCOJ governance policy themes that covered eHealth and EHR implementation within municipalities and specialist care. We abstracted thematic issues related to governance, top-down steering, bottom-up governance, horizontal governance, hierarchical steering, network governance, legal requirements, and funding mechanisms. Issues related to trust, the model’s legitimacy, stakeholders’ autonomy, transparency, and stakeholder participation in the national governance model were also of particular interest.

This data synthesizing process and extraction of policy themes from the OCOJ policy process unfolded the perspectives of the different stakeholders for each theme.

### Ethical Considerations

This project was approved by the data protection officer of the University Hospital North Norway on April 13, 2021 (number 02705; Governance of eHealth: models and strategies to obtain health policy goals—Implementation capacity in a multistakeholder environment).

## Results

### A National Policy Process Characterized by 3 Phases

#### Overview

**Figure 2:**
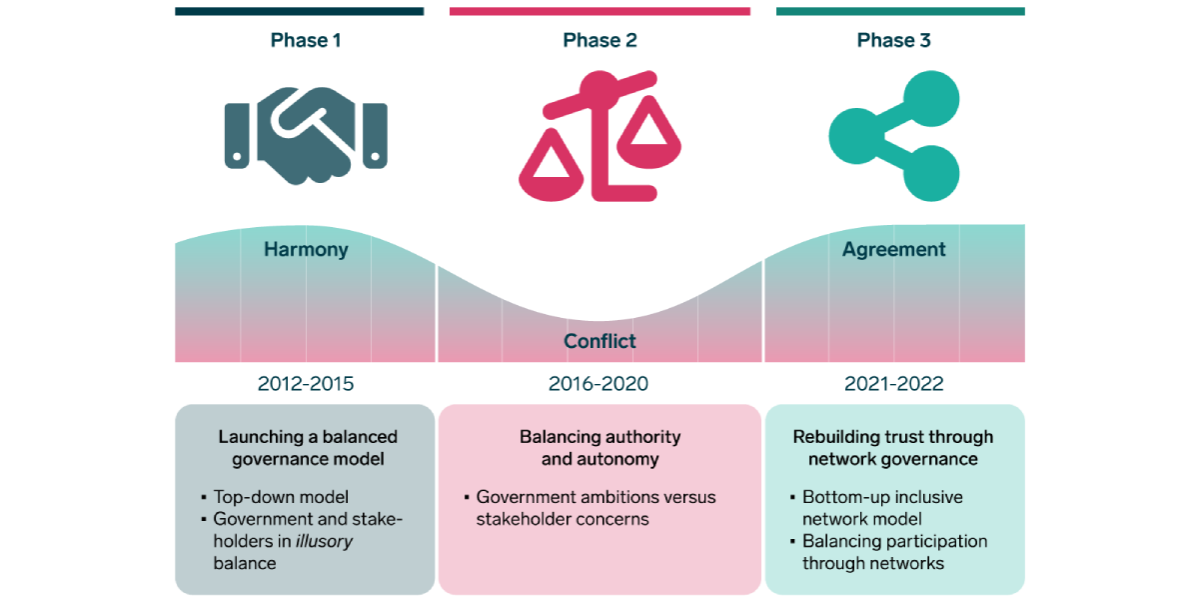
Different phases of the One Citizen–One Journal’s policy process. Each phase is a result of the tension and negotiations between the government and the stakeholders.

We found that the OCOJ policy process unfolded as a consultation process in 3 phases, characterized by distinct empirical turning points (Figure 2). We briefly describe these phases and subsequently categorize the available documents accordingly. Table 3 provides an overview of the stakeholders’ main policy concerns related to policy issues during the various phases (Figure 2).

**Table 3:** Phases, government ambitions, and stakeholders’ concerns.

Policy aim stakeholders	Phase 1: launching a balanced governance model	Phase 2: balancing authority and autonomy	Phase 3: rebuilding trust through network governance
Regional health trusts	Initial support. The regional health trusts lost trust in the NEGB^a^ when the government planned for the Directorate of eHealth to lead, posing a threat to their autonomy.	Ran their own regional EHR^b^ implementation processes independent of the NEGB but promised to coordinate with the NEGB and remain NEGB members	Ran their own regional EHR processes and promised to coordinate with the network governance model
Municipalities or KS^c^	Supported a balanced national governance model and the need for national funding. The municipalities wanted to participate in the process.	Supported the NEGB until they realized that the government was not funding a national EHR. The NEGB did not fulfill its participation and transparency responsibilities. The Directorate of eHealth leadership threatened their autonomy.	Wanted a national governance model based on bottom-up networks that coordinated and governed the municipal “voice” in the market dialogue and in consultations with the government.
Labor unions or organizations of health care professionals	Supported an inclusive national governance model. They expressed a need for national funding and new regulations. They wanted to participate in the national EHR process and the NEGB.	They viewed the implementation of 1 system as too risky. The new EHR systems must be ecosystems that satisfy users’ needs. The NEGB did not contribute to an inclusive horizontal governance model that also included medical governance.	The bottom-up network model would be in line with realizing a national ecosystem that is based on health care workers’ needs and align with bottom-up medical trust needs.
Patient organizations	Supported a top-down inclusive national governance model to increase patients’ access to equal services. Wanted an EHR system that would facilitate easy access to patients’ data.	Supported the NEGB and a strong role for the Directorate of eHealth. This would ensure equal access and quality and strengthen patients’ self-governance of their health data in national health data systems, such as EHRs.	No data
Industry vendors	Supported a model to create bottom-up processes to implement a national network of regional EHRs.	The national governance model did not include all stakeholders to build trust, depriving stakeholders of the opportunity to realize innovation.	No data

^a^NEGB: National eHealth Governance Board.

^b^EHR: electronic health record.

^c^KS: Norwegian Association of Local and Regional Authorities.

#### Phase 1: Launching a Balanced Governance Model (January 2012 to November 2015)

The first phase started with widespread anticipation and support for an inclusive horizontal governance model, but latent tension simmered between the government and stakeholders’ ideas of what an inclusive horizontal governance model would entail in practice [4]. The tension involved stakeholders’ concerns about how the model would balance their autonomy and government ambitions [14,15]. According to the consultation documents, the stakeholders expected the governance model to facilitate participation in a transparent process, along with national funding schemes and legal requirements to support EHR implementation (Textbox 1). The Norwegian Pharmacy Association emphasized the need for both public and private actors’ participation and involvement in the project [58]. The Norwegian Medical Association highlighted the importance of a national funding scheme [56].

When the regional health trusts withdrew from the national project (led by the Directorate of eHealth), the process changed significantly, and phase 2 began [15]. The regional health trusts “did not want a common national EHR solution that included both primary and secondary care because this direction pointed toward a big standard solution” [14]. They withdrew from the national OCOJ project and continued their regional EHR implementation processes [14]. Stakeholders perceived sustained engagement in the national EHR implementation process, overseen by the Directorate of eHealth, as posing challenges to the balance between government authority and stakeholders’ autonomy. This stemmed from the fact that both the regional health trusts and the Directorate of eHealth reported directly to the Ministry of Health and Social Care. Notably, the ministry, as proprietor of the regional health trusts, wielded overarching authority, while the directorate operated as a ministerial agency devoid of direct formal jurisdiction over the regional health trusts [14] (Table 3).

#### Phase 2: Balancing Authority and Autonomy (December 2015 to June 2021)

The government had to scale back its OCOJ ambitions (the NEGB and national EHR project) for phase 2 [15], emphasizing that it is “not appropriate to focus on the choice of one concept alternative in the traditional sense, but one direction of development” [15]. Thus, the government’s main ambition for phase 2 was to realize a national municipal EHR journal and coordinate the process with regional health trusts to attain OCOJ goals [15].

Although the regional health trusts left the national EHR project, they remained members of the NEGB and continued to inform NEGB members about their regional processes [44]. The general feeling toward the NEGB at the beginning of this phase was positive [14]. However, over time, the latent tension between hierarchical and inclusive horizontal governance became evident [14]. The NEGB’s aim was challenged, significantly impacting the goal of realizing an inclusive horizontal governance model based on consensus [4]. The stakeholders became increasingly concerned about the government’s choice of policy actions and how the NEGB balanced top-down authority and stakeholders’ autonomy [14,45,46,52,59,60]. Both the regional health trusts and municipalities acknowledged that “it is demanding to achieve a consensus-based governance model with different member concerns, experiences, and control lines” [14]. To achieve consensus and increase trust in the model, several stakeholders requested that “official hearings should be used to a greater extent than current practice suggests” [14]. No official hearings to elicit feedback on the policy documents issued by the Directorate of eHealth were held between March 2013 and April 2019 [14,46,59] (Tables 1 and 2).

By the end of phase 2, the government had introduced the eHealth Act proposal [16]. Stakeholders’ (eg, regional health trusts, municipalities, and industry organizations) principal concerns focused on how this legislation would formalize the Directorate of eHealth’s dominant position and the NEGB’s function as the steering mechanism for implementing OCOJ policy.

Patient organizations supported the Directorate of eHealth’s dominant role and the NEGB’s role as the steering group. They argued that today’s EHR systems are too fragmented, and patients’ health information would be scattered among different systems without centralized access [61]. They supported the proposed eHealth Act, which, in their view, would function as a tool to improve the digitalization of the domain and strengthen patients’ self-governance of their health data [61].

The Ministry of Health and Social Care stated that by the end of phase 2, cooperation between the government and municipalities was facilitated mainly by traditional hierarchical governance structures outside of the NEGB, such as the Consultation Scheme [55].

Phase 2 ended in June 2021 because of the report from the Office of the Auditor General of Norway that spotlighted deficiencies in the Ministry of Health and Social Care’s governance of the process dating back to January 2012 [14]. The report emphasized that the Ministry of Health and Social Care “has not effectively fulfilled its responsibility for follow-up, quality assurance, and reporting” [14]. Following this critical report and the municipalities’ bypassing of the NEGB, the Ministry of Health and Social Care initiated an evaluation of the governance model [47]. This evaluation resulted in a new inclusive governance model based on a bottom-up network [47].

#### Phase 3: Rebuilding Trust Through Network Governance (July 2021 to December 2022)

Phase 3 was characterized by government efforts to restore confidence in the inclusive governance model. The emphasis shifted to a governance model that would facilitate network governance, to align eHealth governance networks horizontally and hierarchically, with an emphasis on bottom-up processes [48,55]. The government’s focus was primarily on how the revised governance model could be adapted to municipal needs by building on municipal networks [47].

On the basis of the municipalities’ concerns, the new model in phase 3 combined bottom-up network governance with hierarchical governance through the consultation scheme [55]. This combination would ensure that contested topics could be escalated from the network model to the consultation scheme. The network model was accompanied by new procedures to reduce conflict, build trust, and address governance dilemmas [47].

### Two Contrasting Views Regarding Stakeholders’ Autonomy and Top-Down Government Authority

The second main finding is the tension that developed between the 2 contrasting views regarding stakeholders’ autonomy and top-down government authority. Regional health trusts, municipalities, health care professional organizations, and industry actors became increasingly concerned about the model’s ability to balance stakeholders’ autonomy concerns with top-down government authority. In contrast, patient organizations wanted a hierarchical model to ensure equal access to care and quality of care through coherent digital solutions [14,57].

Furthermore, we found that the government and the municipalities disagreed on whether municipal participation in the national EHR project should be mandatory. According to the municipalities, compulsory participation diminished their autonomy while accentuating the hierarchical aspect of the NEGB, rather than fostering its horizontal inclusivity and cooperation potential [60].

Conversely, patient organizations advocated for a “strong governance model” along a different trajectory [61]. The National Umbrella Organization for Patient Organizations supported the proposed eHealth Act and emphasized that “it is necessary for the Directorate of eHealth to have a clear authority role” [59]. From the patient organizations’ perspective, the proposed eHealth Act elucidated and mandated top-down national governance and development requirements to achieve national coherent eHealth solutions [61,62].

Furthermore, our study also found that the patient organizations emphasized the following: “The Norwegian healthcare sector exhibits a complex and fragmented organizational structure. Patients, relatives, and citizens experience firsthand the consequences of inadequate coordination, deficient information flow, and disjointed services and treatment processes. A national governance model and the development of digital services are imperative for patients and citizens, both in terms of patient safety, treatment quality, and the efficient utilization of time and resources” [61]. In the context of a fragmented health care service, ensuring access to up-to-date information is imperative for maintaining a secure patient trajectory [61].

Our findings also show that the municipalities’ apprehension regarding the Directorate of eHealth and NEGB’s commitment to respecting their autonomy prompted them to circumvent the NEGB as a governance tool for the OCOJ initiative, reverting instead to traditional hierarchical governance through the consultation scheme [14].

In its report, the Office of the Auditor General of Norway claimed that the policy became increasingly challenging for “the actors’ autonomy” [14]. The municipalities were concerned that a dominant Directorate of eHealth would impose increased costs for EHR implementation processes [60,63]. However, the municipalities stayed with the process longer than the regional health trusts and supported the NEGB because they expected a national EHR funding scheme [14].

### Participation, Transparency, and Trust as Key Aspects of a Legitimate Inclusive Governance Model

Third, our research revealed that the ambiguity surrounding a national funding scheme and the proposed eHealth Act seemed to escalate tensions between the government and various stakeholders, including municipalities, the Norwegian Medical Association, and industry organizations. Consequently, this heightened tension resulted in greater scrutiny of the transparency and legitimacy of the top-down defined inclusive governance model [14,64]. This was evident in the Norwegian Medical Association’s concerns about the eHealth Act. The Association asserted that the existing governance model and its decision-making structure had become excessively “bureaucratized,” characterized by limited transparency in decision-making processes. Furthermore, the association highlighted a proliferation of issues being raised without full consideration of substantive input [59].

The municipalities and the Norwegian Medical Association increasingly criticized the NEGB’s lack of legitimacy as a governance tool, particularly its lack of ability to facilitate participation, transparency, and trust through an inclusive horizontal governance model [59,60]. Hence, the municipalities circumvented the NEGB based on arguments related to transparency and trust [14].

The lack of official hearings on the reports produced by the Directorate of eHealth contributed to a decrease in their trust in the NEGB [14]. The Norwegian Medical Association stated that the model engendered a perception of “mock processes for numerous individuals within the healthcare sector, contributing to an eHealth environment marked by reduced trust among healthcare personnel” [59]. The association viewed bottom-up network governance as a more suitable way of aligning medical concerns and technological developments in an inclusive model [60,65]. Moreover, our investigation uncovered that the regional health trust of Northern Norway perceived the NEGB as a mechanism wielded by the Directorate of eHealth to justify their top-down policy approach, rather than as a legitimate inclusive horizontal governance model [14].

We found that in its evaluation report, the Directorate of eHealth emphasized that the future governance model (phase 3) “is an advisory arena where the sector gives its recommendations on strategic eHealth issues” [47]. It also emphasized that the model should include all key stakeholders [48]. The bottom-up, inclusive network governance model (in phase 3) combined horizontal and hierarchical governance through the consultation scheme [47]. According to the Directorate of eHealth, the hierarchical aspect of the new inclusive network governance model would bolster its resilience in cases of disputes between stakeholders and the government [47].

Our study showed that in the autumn of 2020, KS assumed leadership and issued a mandate for the national EHR project following consultations with the Directorate of eHealth, the Ministry of Health and Social Care, and participating municipalities [14,55]. The regional health trusts continued their regional implementation processes and promised to align them with the national municipal EHR project [14].

## Discussion

### Principal Findings

The empirical analysis revealed 3 main findings. First, the national policy process evolved across 3 phases, with changes in stakeholder inclusion, participation in the process, and perceived influence on the decision-making process characterizing the transitions. The process commenced with the initial endorsement of an inclusive horizontal governance model, which subsequently was accompanied by stakeholders’ reduced trust in the model. This erosion of trust led to heightened polarization between the government and a pivotal group of stakeholders. Ultimately, the culmination of these developments prompted a re-evaluation of the governance model in favor of a more bottom-up, inclusive network approach. Our second primary finding was the presence of 2 contrasting perspectives regarding stakeholders’ autonomy and top-down government authority related to EHR implementation. Regional health care trusts, municipalities, health care professional organizations, and industry stakeholders expressed growing apprehension about the top-down defined governance model’s capacity to align stakeholder concerns regarding their autonomy to implement their EHRs. Conversely, patient organizations welcomed a top-down hierarchical model to guarantee equitable access, and quality of care through coherent national digital solutions. Our third finding pertained to stakeholder perceptions of government insensitivity concerning participation in the inclusive governance model, the transparency of the model’s decision structure, and its ability to build trust between government and stakeholders. The distrust that emerged between the government and numerous stakeholders posed a challenge to the top-down defined, inclusive governance model. Tension and criticism, fueled by perceived deficiencies in participation, transparency, and trust, made the government change its approach to a bottom-up network model that integrated both inclusive horizontal and hierarchical decision-making.

### A Policy Process Characterized by 3 Phases

This first finding relates to the process unfolding across 3 phases and how this development characterized the process and influenced the inclusive governance model’s ability to address governance dilemmas. The turning point for each phase resulted from stakeholders’ perceptions of participation in and influence on the decision-making process and the governance model’s ability to align these concerns with government ambitions. The phases were characterized by increased stakeholder concerns about lack of participation, transparency, and trust in the OCOJ decision-making process (Figure 2).

The transitions between phases are in line with the findings from New Zealand and Denmark [8,10,11]. A recent review suggested that transitions between phases could be described as “negotiation processes” between government and stakeholders [2]. However, international findings neither delineate the specific stakeholders involved in various phases nor elucidate how governance models in Denmark and New Zealand addressed governance dilemmas regarding stakeholders’ participation to align government objectives with stakeholders’ concerns. In Denmark, a decision was made to adopt a bottom-up municipal network governance approach aimed at fostering dialogue among municipalities. This approach was taken to ensure an equitable process across all municipalities [11]. The government opted for a hands-off approach, diverging from the hands-on approach observed during earlier phases of national EHR projects [11]. This development resembled the Norwegian process, in which municipalities resumed responsibility for the inclusive network governance model following a period characterized by a top-down government approach [11,55].

It is a commonplace observation in public governance research that policy processes unfold in distinct phases [23,28,29]. When policy themes emerge as hot topics, they trigger the mobilization of stakeholders and heighten their demands for participation in the policy process to safeguard their interests [3,23,31]. The tension in policy discussions and negotiations influences how governance models evolve and lead to new phases [23,28,29].

Given that our empirical findings, theoretical perspectives, and international experiences illustrated that policy processes, such as the OCOJ initiative, typically progress through phases, it is reasonable to expect that stakeholders will articulate demands for a more robust and inclusive governance model in the Norwegian eHealth domain in the future, in line with the findings from Denmark and New Zealand [10,11].

### Two Contrasting Views Regarding Stakeholders’ Autonomy and Top-Down Government Authority

The second finding pertains to the 2 divergent stakeholder views regarding how the inclusive governance model would reconcile stakeholders’ autonomy with top-down government authority [14,59]. Patient organizations endorsed a governance model that curtailed regional health trusts’ and municipalities’ autonomy to implement EHRs [61], whereas the regional health trusts, municipalities, and the Norwegian Medical Association supported a bottom-up network model to protect their autonomy and their own EHR implementation processes [14,48] (Figure 1).

We found similar contrasting views in a study from the United Kingdom on radiology networks and a national eHealth project. Radiology stakeholders in the United Kingdom argued that national scaling must balance their medical autonomy against top-down government authority regarding the national standardization of services [66]. They anticipated that the new national EHR project—the National Program for Information in the National Health Service would build on their bottom-up governed medical network [67]. The government did not meet these expectations, leaving radiologists to question how their medical autonomy was met by a top-down hierarchical approach leading to reduced medical trust in national policy [67].

Studies from Denmark are in line with our findings [1,11]. In these studies, the 2 contrasting views were represented by the government and the regions. Evidence revealed government apprehension regarding potential divergence in regional trajectories of EHR implementation, which could result in disparate levels of care access and quality. Consequently, the Danish government opted to use a top-down approach in response to these concerns [2,11]. During different phases, regions of Denmark sought a model that would support their greater autonomy, while at other junctures, the government found it imperative to bolster its top-down governance to align regional and national EHR policies in an inclusive model [11]. Diverging from our comprehensive analysis, which meticulously explored the multifaceted viewpoints of stakeholders, the 2 Danish studies predominantly focus on elucidating the perspectives solely of the regions and the government [1,2,11].

A third study on the Danish eHealth policy process had a similar focus to our study: exploring the national eHealth policy process in a fragmented system. A comprehensive examination of eHealth policy within the Danish context reveals the paramount significance attributed to the national eHealth policy among diverse stakeholders. The dynamics characterizing the interplay between these stakeholders and governmental entities are akin to a strategic power struggle, underscored by varying degrees of power and influence wielded by each stakeholder group [5]. In contrast to our study, this study fails to elucidate whether disparate stakeholder groups harbor divergent perspectives that may be antithetical and how these potential divergent views may have influenced the Danish inclusive governance model [5].

Our study shows that the patient organizations supported a governance model that curtailed the autonomy of regional health trusts and municipalities to ensure easy access to and overview of their health data [59,62]. There is little knowledge of how patient organizations, based on their members’ production of health data, collectively mobilize to exert influence upon the national governance model [2]. A study on mobile apps and sleep monitoring showed that the growth of mobile apps designed to facilitate health and wellness self-governance has generated large amounts of health data, which individuals subsequently may use as a foundation for self-governance to guide their actions [9]. This study does not elaborate on how patient’s ownership of health data may represent a bargaining power in future governance models. However, the study questions how this development may change the patient’s role and influence health care systems [9]. This is in line with the health data governance principles, which aim to create both regulations and international principles that empower the patient’s ownership of their health data in health systems built on human rights [68].

In the “government paradigm,” the government is not the only actor that attempts to influence policy processes and must find ways to govern through stakeholder networks’ dynamics [3,23-25]. In the realm of public governance research, it is commonly observed that governments use various top-down or bottom-up governance strategies to engage in negotiations with heterogeneous stakeholders holding divergent perspectives to cultivate inclusive horizontal governance models [23-25]. In multistakeholder fields, such as eHealth, participating in negotiations and governance models is optional [23,25,28]. If the stakeholders perceive that their goal of preserving their autonomy is overruled or ignored, they may circumvent the governance model or withdraw to their autonomous path to realize their EHR policies. This is in line with our findings. The regional health trusts withdrew from the process, the municipalities circumvented the NEGB, and the Norwegian Medical Association aligned themselves with the KS to increase their medical autonomy, leaving the patient organizations as the strongest supporters of a top-down approach [14].

Our empirical findings, theoretical perspectives, and international experiences illustrate that inclusive governance models must accommodate and align varying concerns among stakeholder groups, for example, regional health trusts, municipalities, health professions, and patient groups regions, to become legitimate, inclusive governance models. Furthermore, our findings elucidate personal health data’s evolving nature and their prospective amalgamation with EHR data, engendering novel dynamics at the nexus of bottom-up and top-down governance mechanisms, thereby shaping future inclusive governance models. A potential implication of this progression is the augmentation of citizens’ perspectives on inclusive governance models in the eHealth realm.

### Participation, Transparency, and Trust as Key Aspects of a Legitimate Inclusive Governance Model

Our third finding pertains to how the government’s insensitivity to stakeholder participation, the governance model’s lack of decision transparency, and decreasing trust between the government and stakeholder groups challenged the inclusive horizontal governance model and its legitimacy. The government changed its approach to an inclusive bottom-up network model that combined horizontal and hierarchical decision-making [14].

The previously mentioned study from New Zealand showed how the government implemented a national inclusive governance model called the National Health IT Board to facilitate national data exchange standards and increased adoption of eHealth [10]. The establishment of this board directly, subordinate to the National Health Board, conferred inclusivity upon the model, encompassing all pertinent stakeholders. This proximity to the national health policy level was deliberately chosen to enhance stakeholder trust in the model as a legitimate policy instrument [10]. This aligns with the findings of our study. The Norwegian government endeavored to establish the NEGB as an inclusive board situated closely to the national health policy level, with the overarching objective of fostering trust in the model and affirming its legitimacy [4].

The second study from Denmark showed that the Danish government in collaboration with regions took a similar approach [11]. Their inclusive governance model aimed to facilitate horizontal dialogue among the regions through a governmental hands-off approach [11]. This model exhibits comparable characteristics to the Norwegian bottom-up inclusive network governance model observed in phase 3, as identified in our examination of the OCOJ policy process. The study from Denmark highlights how this dialogue may increase the transparency on the implementation of EHR standards at both regional, state, and European Union levels [11].

In our examination of the OCOJ process, there was no exploration or discourse regarding the conceptualization of the NEGB as a governance model intended to foster network collaboration with other European countries to augment participation and transparency concerning various transborder eHealth policy matters [15]. The Directorate of eHealth investigated how other countries operationalized their eHealth policy [15]. However, this investigation was focused on understanding the national models, rather than evaluating their potential application as transborder policy instruments in turbulent times [15,31]. The Directorate of eHealth’s work on the OCOJ reports was conducted before the COVID-19 crisis [15,46]. Consequently, their focus on the realization of the NEGB did not account for external turbulent disruptions associated with pandemics and conflicts.

Our study showed that the government aimed to increase the medical trust in the NEGB by including the Norwegian Medical Association as a member of the NEGB. A study from New Zealand showed similar findings [8]. The government of New Zealand aimed at increasing the medical trust in the inclusive national governance model by including medical stakeholders in the model [8]. This finding is in line with our findings. At the outset of phase 1, the government did not see the need to include the Norwegian Medical Association as a member of the NEGB. However, this changed by the end of phase 2 when both the Norwegian Medical Association and the Norwegian Nursing Association became members of the NEGB.

A study from Australia pointed out how the implementation of telehealth systems may facilitate immediate communication between patients and health care providers and increase interprofessional trust [66]. Furthermore, the study pointed out how scaling telehealth in Australia might increase the quality of health care and contribute to an inclusive governance model with increased uptake of evidence-based care [66]. This is in line with our findings on medical trust in the NEGB. The Norwegian Medical Association argued that its membership in the NEGB and participation would ensure the inclusion of the medical perspective and thereby increase health personnel’s trust in the NEGB and the OCOJ policy [56,60].

There is little knowledge of governance models and transparency in eHealth decision processes [2]. However, a study identified transparency as a policy issue related to health information and the sale of pharmaceutical products on the internet [69]. The study questions how the quality assurance of the health information related to the sale of pharmaceutical products on the internet is governed. The authors suggested that increased transparency may be achieved through a global internet governance model that involves the World Health Organization and other relevant stakeholders [69].

The review by Ekeland and Linstad [2] questioned how future national governance models in eHealth may balance global market governance and national top-down or bottom-up approaches. Our investigation and analysis on the Norwegian governance model in eHealth did not find that the Directorate of eHealth planned to explicitly use the NEGB as a tool to balance global market governance and national top-down or bottom-up approaches. We found that the Directorate of eHealth after assessing the market concluded that the only vendors suited for the Norwegian system were global market actors.

On the basis of public governance theory, we suggested that constructions of legitimate governance models require balancing top-down authority and bottom-up autonomy to create trust [23,25]. We also illustrated that inclusive governance models in EHR policy processes must facilitate participation by different stakeholder groups and include transparent policy decision processes to cultivate trust, particularly medical trust [2,3,23]. A possible implication from these findings is that future inclusive horizontal governance models must address dilemmas regarding participation and transparency to build trust by combining network and top-down governance [3,25,27]. Achieving success in establishing an inclusive governance model may entail the incorporation of structured procedures and regulations aimed at addressing misperceptions and enhancing resilience in governing stakeholder trust [31,47,55]. Future developments in health data governance may influence how governments rethink and choose to balance top-down and bottom-up strategies with market-oriented governance approaches to create robust inclusive governance models [29-31].

### Implications and Further Research

The research findings in this case study align with reports from other health systems, underscoring the necessity for governmental attentiveness to policy issues such as participation, transparency, and trust to realize national inclusive horizontal governance models. Additional insights from case studies conducted across diverse contexts are imperative to advance our understanding. Further research is needed to elucidate how different contextual conditions influence governments’ abilities to balance authority and autonomy in a policy field such as eHealth with its fragmented decision authority and plural interests.

### Strengths and Limitations

The research team comprises experienced professionals in eHealth and public governance, drawing on expertise from political science, sociology, medical science, and diverse roles in eHealth research. However, field insights provide a unique starting point for our study. We are aware that our roles as experts may bring biases to our research. Long experience may influence our perception and “worldview” [70]. In contrast, policy studies require a broad overview of the field at the outset of the study [41]. We believe that our awareness of our positionality in the field and the interdisciplinary composition of our research team balance our views and potential biases.

A notable strength of our study lies in its comprehensive incorporation of all publicly available government policy documents and stakeholder consultation materials, which addressed policy proposals and considerations. This enabled us to discern the active participation of stakeholders across different phases and to evaluate how their perspectives influenced the government’s capacity to establish an inclusive horizontal governance model.

Determining the optimal search strategy to identify pertinent documents poses a challenge unless researchers possess a comprehensive understanding and overview of the field from the outset. This aspect may have represented a weakness attributable to the researchers’ focus, as the in-depth longitudinal case study exclusively examined the Norwegian context, potentially limiting its generalizability to the processes and challenges encountered by other countries when implementing EHR policies. Nevertheless, this study may offer valuable insights as a meticulously documented case, providing lessons learned and actionable recommendations for countries facing analogous circumstances.

### Conclusions

We conclude that Norway’s OCOJ policy trajectory was characterized by a process that unfolded across 3 distinct phases. Furthermore, the process was characterized by 2 contrasting views regarding stakeholders’ autonomy to govern their EHR implementation processes and the need for top-down government authority to create a national journal. Regional health trusts, municipalities, health care professional organizations, and industry actors became increasingly concerned about the model’s ability to balance stakeholders’ autonomy and top-down government authority. In contrast, patient organizations wanted an inclusive hierarchical model to ensure equal access to care and quality of care through coherent digital solutions. Finally, the policy process was characterized by diminishing trust in the inclusive governance model.

Our study found that measures were taken to include a variety of voices in the NEGB through consultations. However, the different phases illustrated that participation, transparency, and trust dilemmas can occur in a top-down defined inclusive governance model. Such dilemmas require ongoing addressing through vigilance and responsiveness from governmental entities. The responses made the governance model evolve from being a top-down defined inclusive model into a bottom-up network model.

## Appendix

Multimedia Appendix 1 Policy and consultation response documents, phases, and main contributions.

## Abbreviations

EHR: electronic health record
GP: general practitioner
KS: Norwegian Association of Local and Regional Authorities
NEGB: National eHealth Governance Board
OCOJ: One Citizen–One Journal

## Data Availability

The data used in this paper are publicly available on the websites of the Norwegian Parliament [71] and the Directorate of eHealth [72].

## References

[1] Kierkegaard P (2015). Governance structures impact on eHealth. Health Policy Technol.

[2] Ekeland AG, Linstad LH (2020). Elaborating models of eHealth governance: qualitative systematic review. J Med Internet Res.

[3] Ansell C, Sørensen E, Torfing J (2023). The democratic quality of co-creation: a theoretical exploration. Public Policy Adm.

[4] (2012). Én innbygger - én journal. Digitale tjenester i helse- og omsorgssektoren. Stortinget.

[5] Benneke M, Charalambous G, Jensen O, Andrioti D (2018). Policy analysis for eHealth in Denmark. Int J Community Fam Med.

[6] (2005). Fra stykkevis til helt - en sammenhengende helsetjeneste. Regjeringen.no.

[7] Smaradottir BF, Severinsen GH, Steinsbekk A, Berntsen GK (2021). User-centred design of a digital care plan for patients and professionals in cross-organisational teams. Stud Health Technol Inform.

[8] Atalag K (2013). Using a single content model for eHealth interoperability and secondary use. Stud Health Technol Inform.

[9] Williams SJ, Coveney C, Meadows R (2015). 'M-apping' sleep? Trends and transformations in the digital age. Sociol Health Illn.

[10] Park YT, Atalag K (2015). Current national approach to healthcare ICT standardization: focus on progress in New Zealand. Healthc Inform Res.

[11] Kierkegaard P (2015). Interoperability after deployment: persistent challenges and regional strategies in Denmark. Int J Qual Health Care.

[12] Asthana S, Jones R, Sheaff R (2019). Why does the NHS struggle to adopt eHealth innovations? A review of macro, meso and micro factors. BMC Health Serv Res.

[13] Garmann-Johnsen NF, Eikebrokk TR (2017). Dynamic capabilities in e-health innovation: implications for policies. Health Policy Technol.

[14] Undersøkelser av IT-satsingen Én innbygger ─ én journal; styring og anskaffelser. Riksrevisjonen.

[15] (2014). Utredning av «en innbygger – én journal». Riksrevisjonen.

[16] (2019). Prop. 65 L (2019–2020): lov om e-helse (e-helseloven). Regjeringen.no.

[17] Knutsen O (2017). The Nordic Models in Political Science: Challenged, But Still Viable?.

[18] Pejanovic M, Fischer M (2006). Local self-government: a must for democracy, civil society and EU integration. Peacebuilding and Civil Society in Bosnia-Herzegovina. Ten Years after Dayton.

[19] Laegreid P, Opedal S, Stigen IM (2005). The Norwegian hospital reform: balancing political control and enterprise autonomy. J Health Polit Policy Law.

[20] Tjerbo T (2009). The politics of local hospital reform: a case study of hospital reorganization following the 2002 Norwegian hospital reform. BMC Health Serv Res.

[21] Opedal S, Rommetvedt H (2010). From politics to management – or more politics?. Public Manag Rev.

[22] (2021). Consultations between Central Government and KS. Regjeringen.no.

[23] Ansell CK, Torfing J (2016). Handbook on Theories of Governance.

[24] Jessop B (1997). Capitalism and its future: remarks on regulation, government and governance. Rev Int Polit Econ.

[25] Klijn E (2008). Governance and governance networks in Europe. Public Manag Rev.

[26] Røiseland A, Vabo SI (2008). Governance på norsk. Samstyring som empirisk og analytisk fenomen. Norsk Statsvitenskapelig Tidsskrift.

[27] Osborne SP (2006). The new public governance?. Public Manag Rev.

[28] Morse RS, Stephens JB (2018). Teaching collaborative governance: phases, competencies, and case-based learning. J Public Aff Educ.

[29] Bouckaert G, Im T (2024). Neo weberian state: from theory to practice?. J Policy Stud.

[30] Torfing J (2023). Rethinking Public Governance.

[31] Ansell C, Sorensen E, Torfing J, Trondal J (2024). Robust Governance in Turbulent Times.

[32] Egeberg M, Trondal J (2018). An Organizational Approach to Public Governance: Understanding and Design.

[33] Stoker G (2019). Governance as theory: five propositions. Int Soc Sci J.

[34] Christensen T (2012). Post-NPM and changing public governance. Meiji J Polit Sci Econ.

[35] Koppenjan E, Klijn EH (2015). Governance Networks in the Public Sector.

[36] Braithwaite V, Levi M (1998). Trust and Governance.

[37] Lane C, Bachmann R (1998). Trust Within and Between Organizations: Conceptual Issues and Empirical Applications.

[38] Rossman GB, Rallis SF (2016). An Introduction to Qualitative Research: Learning in the Field.

[39] Walsham G (2017). Interpretive case studies in IS research: nature and method. Eur J Inf Syst.

[40] Appleton JV, King L (2002). Journeying from the philosophical contemplation of constructivism to the methodological pragmatics of health services research. J Adv Nurs.

[41] Brinkmann S, Tanggaard L (2012). Kvalitative Metoder: Empiri og Teoriutvikling.

[42] Basit T (2010). Manual or electronic? The role of coding in qualitative data analysis. Educ Res.

[43] Yin RK (2013). Validity and generalization in future case study evaluations. Evaluation.

[44] Én innbygger – én journal. ehelse.

[45] (2018). Veikart for realiseringen av målbildet for Én innbygger – én journal. Direktoratet for e-helse.

[46] Konseptvalgutredning: nasjonal løsning for kommunal helse- og omsorgstjeneste. Direktoratet for e-helse.

[47] (2022). Videreutvikling av nasjonal styringsmodell for e-helse: evaluering og anbefalinger. Direktoratet for e-helse.

[48] Nasjonal styringsmodell for e-helse. ehelse.

[49] (2019). Nasjonal e-helsestrategi 2017–2022. Direktoratet for e-helse.

[50] (2020). Sentralt styringsdokument: Akson: Helhetlig samhandling og felles kommunal journalløsning. Direktoratet for e-helse.

[51] (2020). Veikart for utvikling og innføring av nasjonale e-helseløsninger 2021 - 2025. Direktoratet for e-helse.

[52] (2018). Kvalitetsrapport: KS1 av nasjonal løsning for kommunal helse- og omsorgstjeneste. Utarbeidet for Finansdepartementet og Helse- Og Omsorgsdepartementet.

[53] (2020). Kvalitetsrapport KS2 av Akson: helhetlig samhandling og felles kommunal journal. Utarbeidet for Finansdepartementet og Helse-og omsorgsdepartementet.

[54] Innstilling fra helse- og omsorgskomiteen om Endringer i pasientjournalloven (tilgjengeliggjøring av og betaling for nasjonale e-helseløsninger m.m.). Stortinget.

[55] Kommunal sektors ambisjoner på e-helseområdet. Kommunesektorens Organisasjon.

[56] (2013). St. Meld. 9 (2012-2013) Én innbygger - én journal. Legeforeningen.

[57] Nowell LS, Norris JM, White DE, Moules NJ (2017). Thematic analysis: striving to meet the trustworthiness criteria. Int J Qual Methods.

[58] Høringsuttalelser og -innspill. Apotekforeningen.

[59] (2020). Videokonferansehøring: lov om e-helse (e-helseloven). Stortinget.

[60] (2019). Felles uttalelse: "en innbygger - en journal". Kommunesektorens Organisasjon and Den Norske Legeforening.

[61] (2020). Høringsinnspill fra Funksjonshemmedes Fellesorganisasjon (FFO). Stortinget.

[62] Én innbygger - én journal. Digitale tjenester i helse- og omsorgssektoren. Stortinget.

[63] (2013). Høringsuttalelse En innbygger ? en journal. The Norwegian Association of Local and Regional Authorities (KS).

[64] (2019). Høring ny e-helselov med endring av forskrift - KS' høringsttalelse. Kommunesektorens Organisasjon.

[65] (2019). Høring - ny e-helselov og endringer i IKT-standardforskriften. Regjeringen.no.

[66] Wade VA, Eliott JA, Hiller JE (2012). A qualitative study of ethical, medico-legal and clinical governance matters in Australian telehealth services. J Telemed Telecare.

[67] Crocker M, Cato-Addison WB, Pushpananthan S, Jones TL, Anderson J, Bell BA (2010). Patient safety and image transfer between referring hospitals and neuroscience centres: could we do better?. Br J Neurosurg.

[68] The principles: health data governance principles. Health Data Governance Principles.

[69] Mackey TK, Eysenbach G, Liang BA, Kohler JC, Geissbuhler A, Attaran A (2014). A call for a moratorium on the .health generic top-level domain: preventing the commercialization and exclusive control of online health information. Global Health.

[70] Darwin Holmes AG (2020). Researcher positionality - a consideration of its influence and place in qualitative research - a new researcher guide. Int J Educ.

[71] Stortinget homepage. Stortinget.

[72] e-helse homepage. e-helse.

