# Supplementary material for: Investigating the Norwegian eHealth Governance Model: Document Study
Source: J Med Internet Res. 2024 Dec 4;26:e59717. doi: 10.2196/59717 (PMC11656114; doi:10.2196/59717)
Supplement: Multimedia Appendix 1 [file jmir_v26i1e59717_app1.docx]

**Multimedia Appendix** Policy and consultation response documents, phases, and main contributions

| **Phase 1:** **Launching a balanced governance model** | | | | |
| --- | --- | --- | --- | --- |
| **Date** | **Document Title** | **Author** | **Status** | **Main contribution** |
| 11/2012 | White Paper No. 9 (2012–2013) One Citizen – One Journal (OCOJ) [1] | Ministry of Health and Care Services | Approved by the government and sent to Parliament | To realize One Citizen – One Journal [i.e., comprehensive, longitudinal electronic health records] in a fragmented system, “a strong national governance model” is needed for coordination, cooperation, and trust. The governance model will create decision transparency and trust among the actors. |
| 11/2012 | Consultation meeting of the Parliamentary Committee of Health and Social Care on OCOJ [2] | Norwegian Medical Association | Sent to Parliament | The Association agrees on OCOJ goals. There is a need for national governance of the eHealth sector and standards that will increase interoperability between the systems. Standards included here are both technological, syntactic, semantic, and organizational. A major increase in funding of ICT investments in the sector also is needed. |
| 1/2013 | Consultation response document on OCOJ [3] | Norwegian Association of Local and Regional Authorities (KS) | Sent to Parliament | KS shares the government’s goals and expects to be included in the process together with KommIT. It also expects a revision of governance and funding mechanisms on the national level. Revision of the governance model may result in either an overly complicated system or a simpler one. KS wants the latter: no more detailed management. |
| 1/2013 | Consultation response document on OCOJ [4] | Norwegian Pharmacy Association | Sent to Parliament | The Association supports a strong national governance model, and long-term funding is needed. |
| 1/2013 | Consultation response document on OCOJ [5] | DIPS ASA | Sent to Parliament | The company [DIPS is a major electronic health record (EHR) vendor, serving three of the four health regions] supports the vision. The process needs to result in a reduction in the number of organizations that share data in a legal way. A joint national municipal journal should have this as a goal. Regional governance is needed because a national journal requires a bottom-up process. |
| 1/2013 | Consultation response document on OCOJ [6] | Norwegian Federation of Organizations of Disabled People (FFO) | Sent to Parliament | Supports the process. A technically similar national journal is needed. Hospital sector funding also is needed. |
| 3/2013 | White Paper No. 9 (2012–2013) [1] | Ministry of Health and Care Services | Approved by Parliament, Innst. 224 S (2012–2013) | Unanimously adopted in Parliament. |
| 12/2015 | Report on OCOJ policy: Executive Summary [7] | Directorate of eHealth | Sent to the Ministry of Health and Care Services | The best concept, which fulfills the main goal of OCOJ,” is a system that includes all actors (i.e., a comprehensive, longitudinal, electronic health record). However, the Directorate abandoned the idea of one system and concluded by emphasizing that this is not a decision on one concept, but on a development direction toward 2040. The hospital regions continue their regional implementation, the Directorate will be responsible for the national municipal journal project, and the National eHealth Governance Board (NEGB) will function as its steering group from 2016. This does not change the lines of control between the actors. |
| **Phase 2:** **Balancing Dependency and Autonomy** | | | | |
| 6/2016 | Report on OCOJ: Evaluating the Health Platform in Mid-Norway as the start of implementation of the OCOJ policy [8] | Directorate of eHealth | Sent to the Ministry of Health and Care Services | The regional implementation project in the Central Norway Health Region, also known as the Health Platform, will be the national pilot of OCOJ. The national project, led by the Directorate, will influence decisions in the regional project the Health Platform to ensure coordination among the other regions and the national joint municipal project. Topics will be discussed in the NEGB. Funding is needed for the national project from 2017. |
| 1/2017 | National eHealth Strategy 2017–2022 [9] | Directorate of eHealth | Sent to the Ministry of Health and Care Services | The national eHealth governance model is a co-governance tool to coordinate and govern the sector in the same direction. The establishment of a national solution provider will contribute to goal attainment. Implementation of a national funding scheme is a prerequisite for the implementation of the national eHealth strategy. |
| 1/2018 | Roadmap for realizing the OCOJ policy [10] | Directorate of eHealth | Sent to the Ministry of Health and Care Services | To realize the OCOJ policy goals, the hospital regions continue their implementation processes. In 2018, the Directorate defined different scenarios for a joint national municipal journal. The report concluded that conditions for the national project need to be defined, such as the national project’s governance model, funding scheme, and legal requirements. |
| 7/2018 | Concept Selection Study on One Joint National Municipal Journal [11] | Directorate of eHealth | Sent to the Ministry of Health and Care Services | This concept study utilizes a scenario methodology to define different scenarios as a basis for parliamentary decisions on national infrastructure projects. To prepare for national investment in OCOJ,” the Directorate conducted a concept study. The primary goal was a concept with a high degree of interoperability, in which the data follow the patient trajectory. C0 represents the status quo. The report defines eight concepts with different degrees of goal attainment and costs. C7 is the concept that the Directorate proposes as the most realistic. All other concepts are compared with C0. C7 defines one national journal for all actors (health providers). It is mandatory that all actors in the municipal care service be part of the national journal. C7 is viewed as having the highest goal attainment and socioeconomic profitability. |
| 12/2018 | Quality assurance report KS1: National solution for municipal health service – one journal [12] | Holte Consulting | Sent to the Ministry of Health and Care Services | To ensure a complete introduction to all municipalities in all regions (except the pilot region of Central Norway), there should be a high degree of commitment from the actors before a major measure can be initiated. The report recommends that the project realize mutually binding agreements with municipalities’ community care groups, GPs, and other private actors during the pre-project phase. Compulsory participation also should be considered during the pre-project phase. C7 assumes 100% participation from contracting parties. Responsibility for the pre-project phase is assigned to the Directorate. The two important issues are: a) the design of the funding model; and b) the national service provider’s mandate should be in place before a final decision on responsibility for the implementation phase. |
| 2/2019 | Joint Statement on further work on the OCOJ common platform for the realization of the vision. [13] | Norwegian Medical Association and KS | Sent to the Ministry of Health and Care Services | KS and the Medical Association agree on the choice of C7. They recommend a flexible implementation process that focuses on innovation and platform development adapted to work processes that facilitate collaboration across organizational borders, including data exchange. They recommend that participation from municipal and private actors be voluntary. Mandatory participation may be an option in the future. The process must be in line with the procurement rules. The development of national eHealth solutions must be based on tight collaboration between healthcare providers and industry vendors. |
| 4/2019 | eHealth Act  Prop. 65 L (2019–2020): The Proposition to Parliament [14] | Ministry of Health and Care Services | Sent to Parliament  Withdrawn from Parliament in 2020 | It is mandatory for the actors in the field to present eHealth projects of national relevance to the Directorate, which will decide whether they should be part of the national portfolio of eHealth projects governed by the Directorate through coordination in NEGB. The Norwegian Health Network (NHN) is obliged to deliver National eHealth Services. The Ministry may define a funding scheme that makes it mandatory for the actors to pay a subscription fee to NHN for eHealth solutions. |
| 6/2019 | National Health and Hospital Plan 2020–2023 [15] | Ministry of Health and Social Care | Sent to Parliament | KS established a co-governance board, KommIT, and a multi-level municipal ICT network. The government will utilize this board to govern the process of a national journal. The eHealth Act increases the Directorate’s mandate to govern and coordinate the national eHealth portfolio and board. A funding scheme for the National eHealth Services also will be created. |
|  |  |  |  | During this phase, the government defined a national joint municipal journal project called Akson. No official hearing on the project was held, but it led to heavy activity in both Parliament and in the media. Discussions were particularly concerned with uncertainties around decisions that the government planned to execute, but did not communicate through an official hearing. The uncertainties related to what extent the government would decide to implement a strong top-down steering model or more horizontal governance that would give stakeholders greater autonomy related to EHR implementation. |
| 12/2019 | Consultation response document on eHealth Act with an amendment to regulations – KS' consultation statement [16] | KS | Sent to the Ministry of Health and Care Services | KS supports the need for national governance to attain national eHealth goals, but KS opposes the proposed mandatory subscription fees for National eHealth Services. The national governance model must be supplemented with a consultation scheme in which KS and the government discuss topics related to development of national eHealth solutions and digital cooperation systems. KS does not support this proposal for a new eHealth Act. |
| 1/2020 | Consultation response document on the eHealth Act [17] | Norwegian Nursing Association | Sent to the Ministry of Health and Care Services | The organization supports implementation of National eHealth Services after they have been tested. Nurses are the biggest group affected by eHealth measures. Under the Act, it would be mandatory for the stakeholders to present their eHealth projects with national relevance to the eHealth Directorate. This will increase reuse of ideas nationally. They suggest that NEGB change its name to the National Coordination Advisory Board. The organization is not represented in NEGB today. |
| 1/2020 | Consultation response document on the eHealth Act [18] | Medical Association | Sent to the Ministry of Health and Care Services | The implementation of eHealth requires a better system for national incentives and a clearer funding model. The evidence that supports the assumption that big systems are better than platform/module systems is weak. Critical of the Directorate’s national role and opposes a mandatory subscription scheme for National eHealth Services, as it reduces innovation potential and changes the traditional governance model in a system of self-regulated actors. |
| 1/2020 | Consultation response document on the eHealth Act [19] | FFO, patient, and consumer organizations | Sent to Parliament | The eHealth Act is a good tool for the national governance of eHealth. Instead of withdrawing the act from a parliamentary decision, these organizations, representing patients and citizens, recommend a restructuring of funding mechanisms for the whole sector. |
| 3/2020 | Akson: “Holistic interaction and Joint Municipal Journal solution”: Main report [20] | Directorate of eHealth | Sent to the Ministry of Health and Care Services | Akson is realizing both a national collaboration system that facilitates high interoperability between the actors and procurement of new electronic health records (EHR) in primary care. The Directorate will be responsible for the project’s first phase, which will be divided into two programs: a) Akson collaboration; and b) Akson AS (joint stock company), with the state as a minority shareholder. The Akson collaboration will develop and harness requirements, and Akson AS will acquire the EHR. The report describes the first phase, in which the Directorate is responsible for development of the collaboration platform. |
| 3/2020 | The Medical Association's comments on Akson [21] | Norwegian Medical Association | Sent to the Ministry of Health and Care Services | The Association expresses concerns that are critical of the economic estimates for Akson. The future journal must be based on an ecosystem perspective and must build on today’s existing EHRs. The government and Akson do not focus on the national collaboration solution, which is important, as is the national medication list. Acquisition of one system is too risky. |
| 6/2020 | External quality assurance report on “holistic collaboration and municipal journal” [22] | Holte Consulting | Sent to the Ministry of Health and Care Services | To ensure necessary goal attainment, the main recommendation is to create a national service provider in which the state procures and operates the medical record solution, and all municipalities, GPs, and other contracting parties are required to use the solution. The report notes that the political conditions complicate their main recommendation. If the political conditions remain unchanged, our second recommendation is to continue the project with a municipal service provider and a requirement for a minimum of 85% binding participation from municipalities and GPs before the announcement of procurement. |
| 10/2020 | eHealth Act  Hearing in the Parliament [23] | Norwegian Centre for eHealth Research |  | The Centre is critical of the governance model, which may centralize decisions. Innovation is bottom-up, not through top-down steering. |
| 10/2020 | eHealth Act  Hearing in the Parliament [24] | Norway Health Tech |  | The Act gives all power to a directorate that does not have health actors’ trust, while simultaneously, the proposal deprives health actors of the ability and opportunity to try out new solutions and influence innovation and business development in Norwegian eHealth. Norway Health Tech believes that Parliament should send the Act back to the Ministry of Care Services to commission a better study of the proposal's financial situation consequences and a model that can give municipalities room for testing health solutions and services. |
| 10/2020 | eHealth Act  Hearing in the Parliament [25] | Tekna |  | The association suggests that the Act be returned to the Ministry of Health and Social Care. |
| 10/2020 | eHealth Act  Hearing in the Parliament [26] | Abelia |  | Abelia suggests that the Act be returned to the Ministry. The new roles for NHN and the Directorate make innovation and testing of new technology difficult. The use of an act as a means of forcing governance is a drastic measure, given that better and more effective approaches to goal achievement can be taken. |
| 10/2020 | eHealth Act  Hearing in the Parliament [27] | ICT Norway |  | The proposal assumes that large, national systems are the best, despite experience and research. Its suggestion is undemocratic and goes against the Norwegian governance tradition of not giving one governmental actor too much power. It also reduces possibilities for business and market growth among Norwegian and international actors. |
| 10/2020 | eHealth Act  Hearing in the Parliament [28] | Norwegian Data Association |  | The Association asks Parliament to send the proposal back to the Ministry. It is not a good tool for better governance in the field, giving the Directorate a new role that does not fit into the normal setup in the healthcare system. It also reduces trust. |
| 10/2020 | eHealth Act  Hearing in the Parliament [29] | Association of the Blind |  | The Association asks what ambitions the Ministry has for universal design. New eHealth services must follow the universal design guidelines. The Act must support increased user participation in service development. |
| 10/2020 | eHealth Act  Hearing in the Parliament [30] | Association of the Hearing Impaired |  | The Association asks what ambitions the Ministry has for universal design. New eHealth services must follow universal design guidelines. The Act must support increased user participation in service development. |
| 10/2020 | eHealth Act  Hearing in the Parliament [31] | National Association of Public Health |  | The Association supports the Act and says it is important to patients that it be implemented. The Association agrees with strong top-down governance in eHealth. |
| 10/2020 | eHealth Act  Hearing in the Parliament [32] | Norwegian Cancer Society |  | The Association supports the proposal, but realizes that it will not be implemented, which is a pity. Once again, the decisions will affect patients, relatives, and citizens negatively. However, it will benefit the actors (municipalities, hospitals, etc.) because their roles will not change in the fragmented system. |
| 10/2020 | eHealth Act  Hearing in the Parliament [33] | Next of Kin Alliance |  | The Alliance supports the Act. We need it now! |
| 10/2020 | eHealth Act  Hearing in the Parliament [34] | Norwegian Medical Association |  | The Association argues that this Act is a tool to strengthen the eHealth governance model legally, but the Association does not support it because the governance model does not work the way it was intended. The Association suggests that the Act be sent back to the Ministry. |
| 10/2020 | eHealth Act  Hearing in the Parliament [17] | Norwegian Nursing Association |  | The Association supports the Act, but would like more assessment on how the national governance model would govern the actors (municipalities, hospitals, private actors, etc.) who are self-governed. Happy to be a member of NEGB. |
| 10/2020 | eHealth Act  Hearing in the Parliament [35] | Norwegian Union of Municipal and General Employees |  | The group does not support the Act and wants to become a NEGB member. The organization does not think the Directorate includes it in policy processes. This must be improved. |
| 10/2020/ | eHealth Act  Hearing in the Parliament [36] | Association of Clinical Nutritionists |  | Working on terminology in various systems is important. |
| 10/2020 | eHealth Act  Hearing in the Parliament [37] | Norwegian Dentist Association |  | Supports the idea of the Act, but is critical of the funding scheme. Dental services are mostly private in Norway. |
| 10/2020 | eHealth Act  Hearing in the Parliament [38] | Consumer Council |  | Supports the Act. There is no time to lose. |
| 10/2020 | eHealth Act  Hearing in the Parliament [39] | FFO |  | The Federation supports the Act. |
| 10/2020 | eHealth Act  Hearing in the Parliament [40] | KS |  | The Association does not support the Act, nor the funding scheme. KS is critical of the Ministry and Directorate functions. These two cannot impose any new funding or priority schemes on municipalities. |
| 10/2020 | eHealth Act  Hearing in the Parliament [41] | Norwegian Society of Engineers and Technologists |  | The organization does not support the Act, but does support increased governance in eHealth. However, it seems like the new governance model (Directorate) will be the watchdog for innovation. NITO does not think this is a good idea. Innovation happens in an open system. The proposal does not describe how prioritization and national funding would work in more detail. |
| 10/2020 | eHealth Act  Hearing in the Parliament [42] | Norwegian Psychological Association |  | Does not support the Act, nor the proposed funding scheme. Its members are often private actors. The organization’s members have not been part of Akson, nor the development of eHealth. This legal proposal will expand the bureaucracy. |
| 11/2020 | Statement from big cities on Akson to the Committee on Health and Social Affairs [43] | Norwegian Association of Local and Regional Authorities (KS) |  | The national municipal journal – we need economic assurance from the Government and risk mitigation before the municipalities can take on full responsibility for the national project. Oslo focuses on “open platforms” – direction for Akson. Increased coordination between municipalities will strengthen them so that they can communicate with the market and speak as one voice. Flexible development with vendors as partners. The Directorate will not be responsible for Akson, and Akson must be better than the alternative and voluntary. |
| 11/2020 | Roadmap for National eHealth Services 2021–2025 (Version 1.01) [44] | Directorate of eHealth | Sent to the Ministry of Health and Care Services | National coordination of the development and implementation of National eHealth Services needs: clearer priorities on what services to implement; a national push for implementation; and national standards. Increased transparency is needed in the National eHealth Services implementation processes. The highest risks for implementation failure are associated with the funding model and system interoperability in primary care services. |
| 6/2021 | Office of the Auditor General's investigation of the Ministry of Health and Care Services' governance of the work on “One Citizen – One Journal”: Attachment 3 to Document 3:14 (2020–2021) [45] | Office of the Auditor General of Norway | Sent to Parliament | The Ministry has not fulfilled its responsibility for follow-up, quality assurance, and effective reporting, which has delayed work on “One Citizen – One Journal.” The Ministry sent ambiguous signals to the Directorate. Uncertainties surfaced as to whether or not the 2015 report should be a concept report, as well as when to start the pre-project for Akson. It was unclear what the funding model would look like, as well as the extent of governmental contributions. Voluntary co-financing did not work out. The actors claim they lack influence on the national governance model. The governance model has failed as an arena for anchoring strategic issues and governmental choice with policy instruments. The government did not offer hearing possibilities on their choices of policy, such as the Directorate’s role as it relates to Akson and the funding. NEGB lacks influence on prioritized actions, such as medication overview. National eHealth priorities happen outside of NEGB. It appears that no connection exists between NEGB and national funding mechanisms. |
| **Phase 3: *Rebuilding trust through network governance*** | | | | |
| 10/2021 | Changes in the Act on the Patient Journal, changes in access to and payment of eHealth solutions, etc., Prop. 3 L (2021–2022), Innst. 47 L (2021-2022), Lovvedtak 26 (2021–2022) [46] | Ministry of Health and Care Services | Sent and decided in Parliament | Funding scheme for national eHealth solutions. The Directorate claims that more legal requirements are needed to build new solutions that will increase electronic collaboration between actors (s*amhandlingsløsning*). The Directorate will define standards and technical requirements, i.e., interoperability. When using the “data infrastructure” concept in the amendments to the Act, the Act facilitates future realization of an interconnected data network that will connect National eHealth Services and patient journals. |
| 12/2021 | Amendments in the legal requirements for national eHealth Solutions [47] | Ministry of Health and Care Services |  | National subscription scheme for national eHealth solutions. The municipalities will be compensated through the national budget. A national technical calculation group will operate as a tool for surveillance of the financial consequences of future national eHealth Solutions. This group is a tool related to the collaboration between the government and KS, and is used to produce documentation for discussions between them. Developments in National eHealth Services and their financial consequences will focus on NEGB, and the collaboration group will be coordinated between KS and the government. |
| 1/2022 | Report on the new revised governance model in eHealth [48] | Directorate of eHealth |  | NEGB changes its name to National Advisory Board in eHealth. Advice is given on strategic measures, such as funding mechanisms, regulations, and organizational aspects on a national level – including all members, not only those from the Directorate. The members are representatives that include government actors, municipalities, hospitals, health professional unions, and patient organizations. Industry vendors are not members. The annual meeting wheel provides an overview of topics in various local, regional, and national eHealth arenas. This will increase transparency. |
| 6/2022 | Summary of consultation response documents on the revised eHealth model [49] | Directorate of eHealth |  | The actors are positive about the governance model and the new name, but they wondered how an advisory board could be more authoritative when it is based on networks. More time needed to anchor processes. The Directorate is not the “leader” of the process like in NEGB. |
| 1/2022 | The Municipal Sector’s Ambitions on eHealth [50] | KS |  | Realization of National eHealth solutions requires a national – and extensive – collaboration between the state and municipalities in a transparent governance model with a sustainable funding mechanism. KS focuses on five areas: 1) citizen services through the national portal Helsenorge.no; 2) a medical overview as part of the care summary; 3) welfare technology; 4) a national journal based on ecosystems and sustainable funding schemes for implementation of municipal EHR; and 5) digitally mediated collaboration. Develop a mutual national governance model (state-municipality) based on horizontal governance. The municipalities need stronger influence on the governance of National eHealth Services. Sustainable funding of the municipality’s costs. Developing local, regional, and national networks to govern portfolios and coordinate on different levels. Coordinate and govern the municipal “voice” in the market dialogue. |
| 3/2022 | Consultation response document on the further development of the national governance model [51] | Norwegian Medical Association | Sent to the Directorate of eHealth | Agrees with the General Auditor that the national governance model has never been a “real governance” model. The changes proposed here will not transform it into a better model, as it cannot address and resolve real challenges. The Ministry and Directorate should be facilitators and coordinators only. The Ministry’s role remains unclear. |
| 3/2022 | Consultation response document on the development of the national governance model [52] | Norwegian Nursing Association | Sent to the Directorate of eHealth | The Association wants representatives from the research community to be members of the eHealth Advisory Council. |
| 4/2022 | Consultation response document on the changes in the Act on the Patient Journal, Prop. 91 (2021-2022), [53] | KS | Sent to Parliament | KS supports the legal changes, which will enhance realization of data exchange and collaboration across organizational borders. It also will support several ongoing activities, including the Health Platform in Central Norway, Joint Municipal Journal, and national collaboration platform. |

Abbreviations:

OCOJ: One citizen – one Jornal

EHR: Electronic Health Record

KS: Norwegian Association of Local and Regional Authorities

Innst: Proposition from the Committee the Parliament

Prop.: Proposition from the Government to the Parliament

References:

1. Ministry of Health and Care Services. Én innbygger - én journal : digitale tjenester i helse- og omsorgssektoren. Oslo, Norway: 2012. Available from: <https://www.regjeringen.no/no/dokumenter/meld-st-9-20122013/id708609/>.

2. The Norwegian Medical Association. Høringsuttalelse St. Meld. 9 (2012-2013) Én innbygger - én journal. Oslo, Norge: 2013. Available from: <https://www.legeforeningen.no/hoeringer/stortinget/2013/5114/hoeringsuttalelse/>.

3. The Norwegian Association of Local and Regional Authorities (KS). Høringsuttalelse En innbygger – en journal. 2013. Available from: <https://www.stortinget.no/no/Saker-og-publikasjoner/Saker/Sak/?p=55703>.

4. The Norwegian Pharmacy Association. Innspill - en innbygger - en journal. 2013. Available from: <https://www.stortinget.no/no/Saker-og-publikasjoner/Saker/Sak/?p=55703>.

5. DIPS ASA. Høring stortingsmelding nr 9, Postion paper on White Paper on One citizen - one Journal. DIPS ASA, 2013. Available from: <https://www.stortinget.no/no/Saker-og-publikasjoner/Saker/Sak/?p=55703>.

6. The Norwegian Federation of Organisations of Disabled People (FFO). Høringsuttalelse Meld St. 9 (2012 - 2013) En innbygger en journal, FFOs merknader til Stortingets Helse- og omsorgskomite. FFO, 2013. Available from: <https://www.stortinget.no/no/Saker-og-publikasjoner/Saker/Sak/?p=55703>.

7. Directorate of eHealth. Utredning av En innbygger en journal sammendrag Oslo, Norway: Directorate of eHealth, 2014-2015. Available from: <https://www.regjeringen.no/contentassets/355890dd2872413b838066702dcdad88/ikt_utfordringsbilde_helse_omsorgssektoren.pdf>.

8. Directorate of eHealth. En innbygger – én journal vurdering av Helseplattformen i Midt-Norge som startpunkt i den nasjonale utviklingsretningen mot realisering av målbildet i Én innbygger – én journal. Oslo, Norway: Direktoratet for e-helse, 2016. Available from: <https://www.ehelse.no/strategi/en-innbygger-en-journal>.

9. Direktoratet of eHealth Nasjonal e-helsestrategi 2017-2022,. Oslo, Norway: 2017. Available from: <https://www.helsedirektoratet.no/rapporter/nasjonal-e-helsestrategi-og-mal-2017-2022/Nasjonal%20e-helsestrategi%202017-2022%20oppdatert%202019.pdf/_/attachment/inline/bb430c3a-5a34-4e6f-a5ba-ed2c8abd7cb7:534d0767482e6ffa21f74ce26839ae21c3d8940b/Nasjonal%20e-helsestrategi%202017-2022%20oppdatert%202019.pdf>.

10. Directorate of e-Health. Veikart for realiseringen av målbildet for Én innbygger –en journal. Oslo. Norway: Direktoratet for e-helse, 2018. Available from: <https://www.ehelse.no/publikasjoner/veikart-for-realiseringen-av-malbildet-en-innbyggeren-journal>.

11. Drectorate of eHealth. En innbygger - en journal Konseptvalgutredning Nasjonal løsning for kommunal helse-og omsorgstjeneste. Oslo: Direktoratet for e-helse, 2018. Available from: <https://www.ehelse.no/tema/Akson/_/attachment/inline/92c02be4-6b21-4a3c-8e91-592ebbef9116:1fc36e2bd732fbc8c69026d259913ebbd3f5c8b8/%C3%89n%20innbygger%20-%20%C3%A9n%20journal%20Konseptvalgutredning%20Nasjonal%20l%C3%B8sning%20kommunal%20helse-%20og%20omsorgstjeneste%20Hovedrapport.pdf>.

12. Holte Consulting. Kvalitetsrapport KS1 av Nasjonal løsning for kommunal helse- og omsorgstjeneste. Utarbeidet for Finansdepartementet og Helse- og omsorgsdepartementet. 2018. Available from: <https://www.ntnu.no/documents/1261860271/1261975586/KS1+av+nasjonal+l%C3%B8sning+for+kommunal+helse-+og+omsorgstjeneste.pdf/556c0998-4fd8-4b7e-9b2e-9323bf62b9c6?version=1.0>.

13. KS and the Norwegian Medical Association. Felles uttalelse: En innbygger - en Journal Det videre arbeidet med En innbygger – en journal – KS og Legeforeningens felles plattform for realisering av visjonen. 2019. Available from: <https://www.ks.no/globalassets/Felles-uttalelse-med-Legeforeningen.pdf>.

14. Ministry of Health and Care Services. Lov om e-helse (e-helseloven) Prop. 65 L (2019 –2020) Proposisjon til Stortinget (forslag til lovvedtak) (eHealth Act). 2019-2020. Available from: <https://www.regjeringen.no/no/dokumenter/prop.-65-l-20192020/id2696053/>.

15. Ministry of Health and Care Services. Meld. St. 7 (2019 – 2020) Melding til Stortinget Nasjonal helse- og sykehusplan 2020 – 2023. 2019. Available from: <https://www.regjeringen.no/contentassets/e353a5d022d84deabd969a5fe043783e/no/pdfs/i-1194_b_kortversjon_nasjonal_helse.pdf>.

16. The Norwegian Association of Local and Regional Authorities (KS). Høringsuttalelse eHelseloven. 2019. Available from: <https://www.ks.no/sok/?query=EHELSELOVEN&filter=alt>.

17. The Norwegian Nursing Association. Høringsuttalelse e-helseloven. Oslo, Norway: The Norwegian Nursing Association, 2020. Available from: <https://www.regjeringen.no/contentassets/2cc94248f15b4838b469a05190941cb1/norsk-sykepleierforbund.pdf?uid=Norsk_sykepleierforbund>.

18. The Norwegian Medical Association. Høringsuttalelse e-helsloven. Oslo, Norway: The Norwegian Medical Association, 2020. Available from: <https://www.legeforeningen.no/contentassets/e94a64d396b445859691331be4666f43/hoeringsuttalelse-ny-e-helselov-og-endringer-i-ikt-standardforskriften-endelig.pdf>.

19. The Norwegian Federation of Organisations for People with Disabilities (FFO) and more. Høringsuttalelse på e-helseloven. Oslo, Norway: 2020. Available from: <https://www.stortinget.no/no/Saker-og-publikasjoner/Saker/Sak/?p=79325>.

20. Directorate of eHealth. Sentralt styringsdokument Akson: Helhetlig samhandling og felles kommunal journalløsning,. Directorate of eHealth, 2020. Available from: <https://www.helsedirektoratet.no/rapporter/sentralt-styringsdokument-akson-helhetlig-samhandling-og-felles-kommunal-journallosning/Sentralt%20styringsdokument%20Akson.pdf/_/attachment/inline/e046726a-e007-44d7-846a-4f8ea1cf74f8:3e21eb92f4e774ccb8e16a83ac41967509ed01ea/Sentralt%20styringsdokument%20Akson.pdf>.

21. The Norwegian Medical Association. Akson sentralt styringsdokument, Akson central steering document,. 2020. Available from: <https://www.legeforeningen.no/fag/ikt/akson/>.

22. Holte Consulting. Kvalitetsrapport KS2 av Akson: Helhetlig samhandling og felles kommunal journal. Utarbeidet for Finansdepartementet og Helse- og omsorgsdepartementet,. Oslo: 2020. Available from: <https://www.regjeringen.no/contentassets/697dd17c89d24b1890d8eb3c511942f7/rapport-ks2-akson.pdf>.

23. Norwegian Centre for E-health Research. Høringsuttalelse e-helseloven. 2020. Available from: <https://www.regjeringen.no/no/dokumenter/horing---ny-e-helselov-og-endringer-i-ikt-standardforskriften/id2675404/?showSvar=true&consterm=&page=1&isFilterOpen=true>.

24. Norway Health Tech. Høringsuttalelse e-helseloven. 2020. Available from: <https://www.regjeringen.no/no/dokumenter/horing---ny-e-helselov-og-endringer-i-ikt-standardforskriften/id2675404/?showSvar=true&consterm=&page=1&isFilterOpen=true>.

25. TEKNA. Høringsuttalelse e-helseloven,. 2020. Available from: <https://www.regjeringen.no/no/dokumenter/horing---ny-e-helselov-og-endringer-i-ikt-standardforskriften/id2675404/?showSvar=true&consterm=&page=1&isFilterOpen=true>.

26. ABELIA. Høringsuttalelse e-helseloven. ABELIA, 2020. Available from: <https://www.abelia.no/arkiv/nyheter5/e-helseloven-vrakes---stortinget-lytter-til-naringslivet/>.

27. ICT Norway. Høringsuttalelse e-helseloven. 2020. Available from: <https://www.regjeringen.no/no/dokumenter/horing---ny-e-helselov-og-endringer-i-ikt-standardforskriften/id2675404/?showSvar=true&consterm=&page=1&isFilterOpen=true>.

28. The Norwegian Data Association. Høringsuttalelse e-helseloven. 2020. Available from: <https://www.regjeringen.no/no/dokumenter/horing---ny-e-helselov-og-endringer-i-ikt-standardforskriften/id2675404/?uid=bce2a094-a3b2-468c-9b6d-436f688c5172>.

29. The Norwegian Association of the Blind and Partially Sighted - NABP. Høringsuttalelse e-helseloven. 2020. Available from: <https://www.stortinget.no/no/Hva-skjer-pa-Stortinget/Horing/horingsinnspill/?dnid=12325&h=10004172>.

30. The Association of the Hearing Impaired. Høringsuttalelse e-helseloven 2020. Available from: <https://www.stortinget.no/no/Hva-skjer-pa-Stortinget/Horing/horingsinnspill/?dnid=12213&h=10004172>.

31. National Association of Public Health. Høringsuttalelse e-helseloven. 2020. Available from: <https://ww>

w.stortinget.no/no/Hva-skjer-pa-Stortinget/Horing/horingsinnspill/?dnid=12276&h=10004172.

32. The Norwegian Cancer Society. Høringsuttalelse e-helseloven. 2020. Available from: <https://www.stortinget.no/no/Hva-skjer-pa-Stortinget/Horing/horingsinnspill/?dnid=12317&h=10004172>.

33. The Next of Kin Alliance. Høringsuttalelse e-helseloven. 2020. Available from: <https://www.stortinget.no/no/Hva-skjer-pa-Stortinget/Horing/horingsinnspill/?dnid=12326&h=10004172>.

34. The Norwegian Medical Association. Høringsuttalelse e-helseloven. 2020. Available from: <https://www.stortinget.no/no/Hva-skjer-pa-Stortinget/Horing/horingsinnspill/?dnid=12316&h=10004172>.

35. Norwegian Union of Municipal and General Employees. Høringsuttalelse e-helseloven. 2020. Available from: <https://www.stortinget.no/no/Hva-skjer-pa-Stortinget/Horing/horingsinnspill/?dnid=12315&h=10004172>.

36. Association of Clinical Nutritionists. Høringsuttalelse e-helseloven. 2020. Available from: <https://www.stortinget.no/no/Hva-skjer-pa-Stortinget/Horing/horingsinnspill/?dnid=12328&h=10004172>.

37. The Norwegian Dentist Association. Høringsuttalelse e-helseloven. 2020. Available from: <https://www.stortinget.no/no/Hva-skjer-pa-Stortinget/Horing/horingsinnspill/?dnid=12266&h=10004172>.

38. The Consumer Council. Høringsuttalelse e-helseloven. 2020. Available from: <https://www.stortinget.no/no/Hva-skjer-pa-Stortinget/Horing/horingsinnspill/?dnid=12324&h=10004172>.

39. The Norwegian Federation of Organisations of Persons with Disabilities (FFO). Høringsuttalelse e-helseloven. 2020. Available from: <https://www.stortinget.no/no/Hva-skjer-pa-Stortinget/Horing/horingsinnspill/?dnid=12318&h=10004172>.

40. The Norwegian Association of Local and Regional Authorities (KS) is the organisation for all local governments in Norway. Høringsuttalelse e-helseloven. 2020. Available from: <https://www.stortinget.no/no/Hva-skjer-pa-Stortinget/Horing/horingsinnspill/?dnid=12314&h=10004172>.

41. Norwegian Society of Engineers and Technologists. ,Høringsuttalelse e-helseloven. 2020. Available from: <https://www.regjeringen.no/no/dokumenter/horing---ny-e-helselov-og-endringer-i-ikt-standardforskriften/id2675404/?uid=c06ca148-d271-4387-8125-3962c34a5cb8>.

42. Norwegian Psychological Association. Høringsuttalelse e-helseloven. 2020. Available from: <https://www.regjeringen.no/no/dokumenter/horing---ny-e-helselov-og-endringer-i-ikt-standardforskriften/id2675404/?uid=38a98f10-df7f-40d7-96a8-39c9c68183e0>.

43. Kristiansand Municipality. Uttalelse fra storbyer om Akson til Helse- og omsorgskomiteen Statement from the big cities on Akson to the Health and Care Committee. Kristiansand Municipality, 2020. Available from: <https://www.ks.no/contentassets/cdc85f2b3ac14f78a75fde4fe5ae611b/Uttalelse-fra-storbyer-om-Akson-til-Helse-og-omsorgskomiteen.pdf>.

44. Direktoratet for e-helse Directorate of eHealth. Veikart for utvikling og innføring av nasjonale e-helseløsninger 2021 - 2026. Oslo, Norway: 2020 - 2022. Available from: <https://www.helsedirektoratet.no/rapporter/veikart-for-utvikling-og-innforing-av-nasjonale-e-helselosninger-2021-2026/Veikart%20for%20nasjonale%20e-helsel%C3%B8sninger-versjon%2022.3.pdf/_/attachment/inline/7f185f6f-e4e8-4a71-8a4e-8c024ae7499c:18b5952b33f7194ac863b0aae932182f95a92822/Veikart%20for%20nasjonale%20e-helsel%C3%B8sninger-versjon%2022.3.pdf>.

45. Office of the Auditor General of Norway. Undersøkelser av IT-satsingen Én innbygger ─ én journal; styring og anskaffelser. 2021. Available from: <https://www.riksrevisjonen.no/rapporter-mappe/no-2020-2021/undersokelser-av-en-innbygger---en-journal-styring-og-anskaffelser/>.

46. Ministry of Health and Care Services. Innstilling fra helse- og omsorgskomiteen om Endringer i pasientjournalloven (tilgjengeliggjøring av og betaling for nasjonale e-helseløsninger m.m.). 2021-2022. Available from: <https://www.stortinget.no/no/Saker-og-publikasjoner/Publikasjoner/Innstillinger/Stortinget/2021-2022/inns-202122-047l/?all=true>.

47. Ministry of Health and Care Services. Prop. 3 L (2021–2022) Endringer i pasientjournalloven (tilgjengeliggjøring av og betaling for nasjonale e-helseløsninger m.m.). Oslo, Norway: 2021. Available from: <https://www.regjeringen.no/no/dokumenter/prop.-3-l-20212022/id2875684/>.

48. Directorate of e-Health. Videreutvikling av nasjonal styringsmodell for e-helse Evaluering og anbefalinger Development of national e-health governance model Evaluation and recommendations,. Oslo, Norway: Directorate of e-Health, 2022. Available from: <https://www.ehelse.no/horinger/horing-nasjonal-styringsmodell-for-e-helse/_/attachment/inline/ebf256b3-c979-46d2-97e3-645ba3e2cd74:f9ee9518e6345bf0e4a73ccafcdec1b12e9db9a0/Rapport%20og%20h%C3%B8ringsdokument_Videreutvikling%20av%20nasjonal%20styringsmodell%20for%20ehelse.pdf>.

49. Directorate of eHealth. Oppsummering av høringssvar til videreutvikling av nasjonal styringsmodell for e-helse. 2022. Available from: <https://www.ehelse.no/horinger/horing-nasjonal-styringsmodell-for-e-helse>.

50. The Norwegian Association of Local and Regional Authorities Kommunal sektors ambisjoner på e-helseområdet Felles plan og rammeverk Municipal sector ambitions on the e-health area Common plan and framework. Oslo, Norway: 2022. Available from: <https://www.ks.no/fagomrader/digitalisering/felleslosninger/digitalisering-i-helse-og-omsorgsektoren-e-helse/kommunal-sektors-ambisjoner-pa-e-helseomradet/>.

51. The Norwegian Medical Association. Høringsuttalelse på den nasjonale eHelse styringsmodellen. 2022. Available from: <https://www.legeforeningen.no/hoeringer/interne/2022/hoering-nasjonal-styringsmodell-for-e-helse/hoeringsgrunnlag/>.

52. The Norwegian Nursing Association. Videreutvikling av nasjonal styringsmodell i eHelse. 2022. Available from: <https://www.ehelse.no/horinger/horing-nasjonal-styringsmodell-for-e-helse>.

53. The Norwegian Association of Local and Regional Authorities. Endringer i pasientjournalloven mv. (nasjonal digital samhandling) Prop. 91 L (2021-2022). 2022. Available from: <https://www.ks.no/horingssvar/horingssvar-endringer-i-pasientjournalloven/>.
